# Serum metalloproteinase-9 is related to COPD severity and symptoms - cross-sectional data from a population based cohort-study

**DOI:** 10.1186/s12931-015-0188-4

**Published:** 2015-02-21

**Authors:** Robert Linder, Eva Rönmark, Jamshid Pourazar, Annelie Behndig, Anders Blomberg, Anne Lindberg

**Affiliations:** Department of Public Health and Clinical Medicine, Division of Medicine, Umeå University, SE-90187 Umeå, Sweden; Department of Public Health and Clinical Medicine, the OLIN unit, Division of Occupational and Environmental Medicine, Umeå, University,, SE-90187, Umeå, Sweden

**Keywords:** Lung function, Productive cough, TIMP-1, MMP-9/TIMP-1 ratio, Proteases

## Abstract

**Background:**

Chronic obstructive pulmonary disease, COPD, is an increasing cause of morbidity and mortality worldwide, and an imbalance between proteases and antiproteases has been implicated to play a role in COPD pathogenesis. Matrix metalloproteinases (MMP) are important proteases that along with their inhibitors, tissue inhibitors of metalloproteinases (TIMP), affect homeostasis of elastin and collagen, of importance for the structural integrity of human airways. Small observational studies indicate that these biomarkers are involved in the pathogenesis of COPD. The aim of this study was to investigate serum levels of MMP-9 and TIMP-1 in a large Swedish population-based cohort, and their association with disease severity and important clinical symptoms of COPD such as productive cough.

**Methods:**

Spirometry was performed and peripheral blood samples were collected in a populations-based cohort (median age 67 years) comprising subjects with COPD (n = 594) and without COPD (n = 948), in total 1542 individuals. Serum MMP-9 and TIMP-1 concentrations were measured with enzyme linked immunosorbant assay (ELISA) and related to lung function data and symptoms.

**Results:**

Median serum MMP-9 values were significantly higher in COPD compared with non-COPD 535 vs. 505 ng/ml (*P* = 0.017), without any significant differences in serum TIMP-1-levels or MMP-9/TIMP-1-ratio. In univariate analysis, productive cough and decreasing FEV_1_% predicted correlated significantly with increased MMP-9 among subjects with COPD (*P* = 0.004 and *P* = 0.001 respectively), and FEV_1_% predicted remained significantly associated to MMP-9 in a multivariate model adjusting for age, sex, pack years and productive cough (*P* = 0.033).

**Conclusion:**

Productive cough and decreasing FEV_1_ were each associated with MMP-9 in COPD, and decreasing FEV_1_ remained significantly associated with MMP-9 also after adjustment for common confounders in this population-based COPD cohort. The increased serum MMP-9 concentrations in COPD indicate an enhanced proteolytic activity that is related to disease severity, and further longitudinal studies are important for the understanding of MMP-9 in relation to the disease process and the pathogenesis of different COPD phenotypes.

## Background

Chronic obstructive pulmonary disease, COPD, is a common chronic disease, characterised by chronic airflow limitation, recurring exacerbations and a range of pathologic changes in the lungs. COPD is described as a heterogeneous syndrome of overlapping conditions such as chronic bronchitis, emphysema and bronchiolitis [1]. Recent studies and guidelines acknowledge the importance of airway inflammation in the process of COPD development [1]. The chronic inflammatory processes in COPD leads to the loss of alveolar attachments to the small airways and decreased lung elastic recoil [2]. In turn, these changes diminish the ability of the airways to remain open during expiration, thus limiting expiratory flow.

Current hypotheses suggest that inflammation, protease-antiprotease imbalance, oxidative stress and accelerated ageing of the lung can be accredited to the pathogenesis of COPD [3]. As part of the protease-antiprotease system, matrix metalloproteinase-9 (MMP-9) has gained an increasing research interest in COPD [4]. MMP-9 is a multi-domain enzyme with many functions in biology and pathology, among which the breakdown of collagen and gelatine is of significance in the pathogenesis of COPD [5-7]. Finding a COPD biomarker easily measured in peripheral blood, is clearly an appealing goal, especially if this biomarker would correlate with measures of disease progression.

In a study of resected human lung parenchyma from 26 patients, MMP-9 expression and the molar ratio of MMP-9 to tissue inhibitor of metalloproteinases-1 (TIMP-1) were increased in smokers compared with non-smokers, and correlated with the burden of cigarette smoking. There was also an inverse association between MMP-9 concentrations and FEV_1_% predicted values [8]. In a Swedish population-based study employing 1,016 subjects aged 70 years, the serum levels of MMP-9 were inversely associated with FEV_1_ without discriminating for obstructive lung function impairment. MMP-9 and TIMP-1 serum levels were also associated with smoking status, being lowest in never-smokers and highest in current smokers [9].

MMP-9 is also thought to play an important role in lung remodelling and has been investigated as a potential biomarker of COPD, given that increased elastolytic activity is a significant part of emphysema [10]. In a study comparing 23 patients with moderate to severe COPD with age-matched controls, serum MMP-9 was negatively correlated with both FEV_1_ and the FEV_1_/FVC ratio [10]. TIMP-1 is also important to consider when studying MMP-9, since it is suggested to inhibit the elastolytic activity of MMPs [11,12]. The relationship between these biomarkers and FEV_1_ among subjects with COPD has so far only been evaluated in fairly small observational studies, resulting in important information regarding the involvement of metalloproteinases in the pathogenesis of COPD. Besides lung function, also bronchitis symptoms and exacerbations are negative prognostic factors and related to disease severity in COPD [13,14], hence important to evaluate in relation to these biomarkers. However, there is a significant under-diagnosis of COPD [15-17] and studies of population-based cohorts are therefor important in order to evaluate the significance of these biomarkers in COPD pathogenesis in general.

The aim of this population-based study was to compare serum MMP-9, TIMP-1 and MMP-9/TIMP-1 ratio in subjects with and without COPD and further to evaluate the association between these biomarkers and factors of clinical significance, such as burden of tobacco smoking, productive cough and lung function among subjects with and without COPD. The hypothesis was that the study setup with COPD subjects and non-COPD subjects as controls would elucidate whether MMP-9 is related to COPD severity in a population based study.

## Methods

### Participants and design

After re-examination of four adult population cohorts from the OLIN-studies (Obstructive Lung Disease in Northern Sweden) during 2002–04, all subjects with COPD were identified together with age and gender-matched controls without obstructive lung function impairment. Since 2005, the study population (n = 1986) has been annually invited to a basic examination program including spirometry and a structured interview [18].

This report is based on data from the examinations in 2005, when blood samples were collected in addition to the basic program. In 2005, 1806 out of 1915 subjects still alive (94%) participated, whereof 164 subjects not able to attend the examinations were interviewed by telephone, 1626 subjects performed spirometry and 1621 participated in the blood sampling. The study population in this paper includes all subjects with complete data from both spirometry and blood samples (n = 1542). The Regional Ethics Committee at Umeå University approved the study, which was carried out according to the declaration of Helsinki.

### Questionnaire

Previously well validated questions regarding respiratory symptoms were used in a structured interview [19-21]. The Modified Medical Research Council Dyspnoea Scale, mMRC, was included in the questionnaire to grade dyspnoea, scale 0–4 [22]. In addition, data on smoking habits and co-morbidities were included.

### Definitions

Body Mass Index (BMI) was calculated: weight (kg)/ (height (m)*height (m)) and was classified as underweight (<20), normal (20– < 25), overweight (25– < 30) and obesity (≥30).

Smoking habits were classified as: non-smokers, ex-smokers (stopped since at least one year) and current smokers. Pack-years were calculated as (number of cigarettes smoked per day × number of years smoked)/20. We defined productive cough as cough and phlegm on most days for at least three months during the last 12 months. Clinically significant dyspnoea was defined as mMRC score ≥2.

### Spirometry and classification of COPD

The lung function tests were performed using a dry spirometer, Mijnhardt Vicatest 5, the Netherlands, following the ATS guidelines [23]. A reversibility test was performed if FEV_1_/highest of FVC or SVC <0.70, or if FEV_1_ < 80% of predicted value. COPD was defined as FEV_1_ divided by FVC or SVC <0.70, using the highest value pre- or post bronchodilatation. Disease severity was classified according to the GOLD (Global Initiative for Obstructive Lung Disease) spirometric criteria based on FEV_1_% predicted; GOLD 1 – 4 [1]. Swedish spirometric reference values were used [24].

### Laboratory analysis

All samples were stored since 2005 at −20**°**C, thereafter thawed and analysed at the same time. Serum concentrations of MMP-9 and TIMP-1 were assayed using the same batch of a commercially available ELISA kit (DuoSet® ELISA Development System, R&D Systems Europe Ltd., United Kingdom) according to the manufacturer’s instructions. Briefly, recombinant human MMP-9 and TIMP-1 were used to construct a standard curve (range 39–2,500 pg/ml) for each set of samples assayed, serum samples were diluted 150 times and concentration read from the standard curve were multiplied by the dilution factor.

### Statistical analysis

Dichotomous variables were analysed using Pearson’s χ2, continuous variables with the independent variable t-test for variables that met the assumption of normal distribution, and the non-parametric Mann–Whitney U-test for those that did not. Non-normal distributed data are presented as median and interquartile range (IQR). Since there were very few subjects in GOLD 3 and 4, they were treated as one group. Univariate analyses of biomarkers (MMP-9, TIMP-1 and MMP-9/TIMP-1 ratio), stratified by COPD and non-COPD, were performed in relation to factors as sex, age, BMI-class, smoking habits, pack years, productive cough and FEV_1_% predicted. The same biomarkers were analysed in linear regression models, stratified by COPD and non-COPD, including the independent variables sex, age, pack years, productive cough and FEV_1_% predicted. The Statistical Package for the Social Sciences (SPSS) version 21.0 for Mac was used for all analyses and P-values < 0.05 were considered significant.

## Results

### Study population

The basic characteristics of the study population (COPD n = 594, non-COPD n = 948) are presented in Table 1. There were significant differences observed in distribution of sex, age and BMI when comparing COPD and non-COPD.

**Table 1:** **Study population characteristics; demographics, smoking habits, respiratory symptoms and biomarker levels, comparing non-COPD and COPD and also presented by GOLD stages**

	**Non-COPD**	**COPD**	**P-value**	**GOLD 1**	**GOLD 2**	**GOLD 3-4**
	**(n = 948)**	**(n = 594)**		**(n = 378)**	**(n = 190)**	**(n = 26)**
Female, n (%)	447 (47)	248 (42)	**0.038**	155 (41)	87 (46)	6 (23)
Age (years), median (IQR)	67 (55–71)	69 (57–71)	**0.015**	69 (56–71)	69 (58–71)	70 (64–79)
BMI (kg/m2), median (IQR)	26.8 (24.3-29.7)	26.1 (23.5-28.7)	**<0.001**	25.8 (23.6-28.4)	26.5 (23.5-29.0)	27.6 (21.7-29.4)
Pack years, median (IQR)	0.8 (0–12)	14 (0–27)	**<0.001**	8.6 (0–24)	18.5 (2.0-32.3)	26.9 (14.8-42.5)
Non-smoker, n (%)	443 (47)	149 (25)	**<0.001**	113 (30)	34 (18)	2 (8)
Ex smoker, n (%)	384 (41)	247 (42)	0.676	153 (40)	75 (41)	19 (73)
Current smoker, n (%)	120 (13)	197 (33)	**<0.001**	111 (29)	81 (43)	5 (19)
Productive cough, n (%)	212 (22)	238 (40)	**<0.001**	125 (33)	93 (49)	20 (77)
mMRC dyspnoea score ≥2, n (%)	46 (5)	75 (13)	**<0.001**	17 (5)	40 (22)	18 (69)
FEV_1_% predicted^†^, median (IQR)	1.03 (0.93-1.13)	0.85 (0.73-0.96)	**<0.001**	0.93 (0.86-1.01)	0.70 (0.64-0.75)	0.44 (0.37-0.47)
MMP-9 (ng/ml), median (IQR)	505 (364–606)	535 (315–653)	**0.017**	513 (241–626)	559 (379–662)	647 (565–692)
TIMP-1 (ng/ml), median (IQR)	316 (229–490)	304 (227–439)	0.252	311 (237–467)	294 (218–425)	298 (247–383)
Ratio of MMP-9/TIMP-1, median (IQR)	1.36 (0.85-2.09)	1.5 (0.83-2.32)	0.168	1.46 (0.78-2.18)	1.48 (0.9-2.5)	1.95 (1.47-2.79)

IQR = Inter quartile range. Significant p-values, p < 0.050, in bold.

### Biomarkers in non-COPD and COPD as well as COPD by GOLD stage

**Figure 1:**
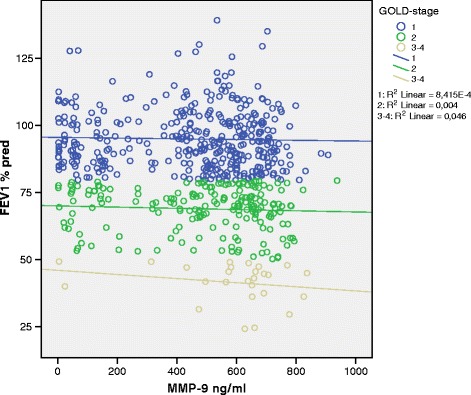
**Association between FEV**_**1**_ **% predicted and MMP-9 in COPD subjects, the lines show the linear association between MMP-9 and FEV**_**1**_ **% predicted in each GOLD stage with the R** ^**2**^ **value indicating the strength of the association.**

**Figure 2:**
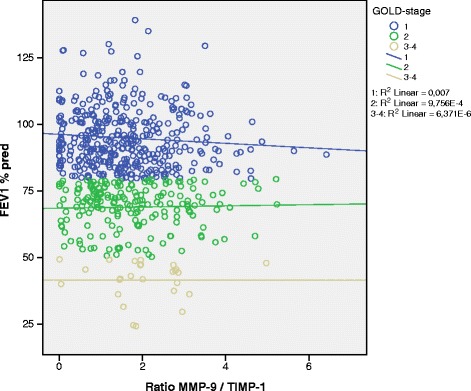
**Association between FEV**_**1**_ **% predicted and the MMP-9/TIMP-1-ratio in COPD subjects, the lines show the linear association between MMP-9/TIMP-1-ratio and FEV**_**1**_ **% predicted in each GOLD stage with the R** ^**2**^ **value indicating the strength of the association.**

Median serum MMP-9 values were significantly higher in COPD compared with non-COPD, 535 ng/ml vs. 505 ng/ml (p = 0.017), whereas there were no significant differences in serum TIMP-1-levels or MMP-9/TIMP-1-ratio (Table 1). There was a tendency towards higher MMP-9-levels with higher GOLD-stage (Table 1). Figure 1 shows the association between MMP-9 values and FEV_1_% predicted in COPD by GOLD stage, each circle representing one subject, whereas Figure 2 shows the association between MMP-9/TIMP-1-ratio and FEV_1_% predicted in COPD by GOLD stage.

### Univariate association with biomarkers in non-COPD and COPD

In univariate analysis, pack years and current smoking were significantly associated with increased MMP-9 in both non-COPD and COPD. In COPD, productive cough and decreasing FEV_1_% predicted were significantly associated with increased MMP-9 (Table 2).

**Table 2:** **Univariate analyses of associations between various factors and MMP-9 in non-COPD and COPD, respectively**

	**Non-COPD**		**COPD**	
	**Beta**	**P-value**	**Beta**	**P-value**
Sex (female^†^)	−0.050	0.122	−0.070	0.087
Age^‡^	0.003	0.928	0.037	0.370
Underweight (normal weight^†^)	0.038	0.512	−0.039	0.560
Overweight (normal weight^†^)	−0.018	0.634	−0.051	0.266
Obese (normal weight^†^)	0.064	0.155	0.022	0.706
Pack years^‡^	**0.096**	**0.003**	**0.115**	**0.005**
Ex-smoker (non smoker^†^)	−0.011	0.744	0.098	0.052
Current smoker (non smoker^†^)	**0.118**	**0.005**	**0.218**	**<0.001**
Productive cough, prc (no prc^†^)	0.040	0.215	**0.119**	**0.004**
mMRC ≥ 2 (<2^†^)	0.029	0.377	0.049	0.238
FEV_1_% pred^‡^	−0.060	0.067	**−0.131**	**0.001**

^†^reference value, ^‡^continuous variable. Significant p-values, p < 0.050, in bold.

Corresponding analyses for TIMP-1 showed that increasing age was significantly related to increasing TIMP-1 in both non-COPD and COPD (Beta: 0.122, p < 0.001 and 0.108, p = 0.009 respectively). Increasing pack years was associated with decreasing TIMP-1 in non-COPD(Beta: −0.074, p = 0.023), while current smoking was associated with decreased TIMP-1 in COPD (Beta: −0.123, p = 0.013). Overweight was associated with increased TIMP-1 only in COPD (Beta: 0.113, p = 0.014).

Increasing pack years and current smoking were associated with increasing MMP-9/TIMP-1-ratio in both non-COPD and COPD. In non-COPD increasing age was associated with a decreasing MMP-9/TIMP-1-ratio, while in COPD, overweight and decreasing FEV_1_% predicted were associated with increasing ratio (Table 3).

**Table 3:** **Univariate analyses of associations between various factors and MMP-9/TIMP-1 ratio in non-COPD and COPD, respectively**

	**Non-COPD**		**COPD**	
	**Beta**	**P-value**	**Beta**	**P-value**
Sex (female^†^)	−0.038	0.245	−0.007	0.874
Age^‡^	**−0.081**	**0.012**	−0.074	0.072
Underweight (normal weight^†^)	0.001	0.992	−0.054	0.423
Overweight (normal weight^†^)	−0.124	0.221	**−0.118**	**0.010**
Obese (normal weight^†^)	−0.009	0.837	0.202	0.840
Pack years^‡^	**0.099**	**0.002**	**0.124**	**0.003**
Ex-smoker (non smoker^†^)	0.045	0.195	**0.102**	**0.043**
Current smoker (non smoker^†^)	**0.106**	**0.011**	**0.256**	**<0.001**
Productive cough, prc (no prc^†^)	0.004	0.904	0.060	0.148
mMRC ≥ 2 (<2^†^)	0.003	0.929	0.034	0.424
FEV_1_% pred^‡^	−0.040	0.219	**−0.118**	**0.004**

^†^reference value, ^‡^continuous variable. Significant p-values, p < 0.050, in bold.

### Multivariate association with biomarkers in non-COPD and COPD

In the multivariate analyses stratified by non-COPD and COPD, MMP-9 remained significantly associated with increasing pack-years in non-COPD and decreasing FEV_1_% predicted in COPD, independent of sex, age and productive cough (Table 4). In a corresponding model, TIMP-1 was associated with increasing age in both non-COPD and COPD (Beta: 0.104, p = 0.002 and 0.102, p = 0.014), while in non-COPD only associated with productive cough and decreasing pack years (Beta: 0.065, p = 0.050 and −0.072, p = 0.034 respectively). Similar analyses of the MMP-9/TIMP-1 ratio revealed a significant association with increasing pack years in both non-COPD and COPD, while the association with increasing age was border-line significant in non-COPD (p = 0.051) as was decreasing FEV_1_% predicted in COPD (p = 0.051) (Table 5).

**Table 4:** **Multivariate analyses of factors associated with MMP-9 in in non-COPD and COPD, respectively**

	**Non-COPD**		**COPD**	
	**Beta**	**P-value**	**Beta**	**P-value**
Sex (female^†^)	0.038	0.256	0.060	0.140
Age^‡^	0.025	0.460	0.032	0.444
Productive cough (no prc^†^)	0.012	0.714	0.078	0.068
Pack years^‡^	**0.084**	**0.013**	0.077	0.069
FEV_1_% pred^‡^	−0.052	0.120	**−0.092**	**0.033**

^†^reference value, ^‡^continuous variable. Significant p-values, p < 0.050, in bold.

**Table 5:** **Multivariate analyses of factors associated with MMP-9/TIMP-1 ratio in non-COPD and COPD, respectively**

	**Non-COPD**		**COPD**	
	**Beta**	**P-value**	**Beta**	**P-value**
Sex (female^†^)	0.021	0.534	−0.007	0.856
Age^‡^	−0.065	0.051	−0.075	0.071
Productive cough (no prc^†^)	−0.009	0.796	0.036	0.400
Pack years^‡^	**0.088**	**0.010**	**0.095**	**0.027**
FEV_1_% pred^‡^	−0.020	0.553	−0.084	0.051

^†^reference value, ^‡^continuous variable. Significant p-values, p < 0.050, in bold.

Adjustments made for BMI-class, on-going or recent infection, cancer diagnosis and oral corticosteroid use did not alter main findings of the multivariate analyses.

## Discussion

In this population-based study, subjects with COPD had significantly higher serum MMP-9 compared to those without obstructive lung function impairment. Among subjects with COPD, MMP-9 was significantly associated with productive cough and decreasing FEV_1_% predicted, and in a multivariate model, decreasing FEV_1_ remained significantly associated with MMP-9, independent of sex, age, pack years and productive cough. In corresponding analyses among non-COPD-subjects only pack years remained significantly associated with MMP-9. Serum TIMP-1 levels were similar in COPD and non-COPD, however, analysis of the MMP-9/TIMP-1-ratio may suggest an imbalance in the pulmonary protease-antiprotease homeostasis among COPD-subject, with a predominance of proteolytic activity.

An association between smoking and MMP-9 has been shown previously [8,9,25]. In the present univariate analyses, current smoking was associated with MMP-9 as well as increased MMP-9/TIMP-1 ratio, suggesting that smoking per se increases the proteolytic activity in both COPD and non-COPD. In this context, it deserves to be mentioned that the effect of smoking on MMP-9 may, at least in sputum, remain at least 6 months after smoking cessation [26]. Furthermore, the burden of tobacco smoke exposure, assessed as pack years, was associated with increasing MMP-9/TIMP-1-ratio in both COPD and non-COPD, indicating a tobacco smoke-induced increase in proteolytic activity, independent of sex, age and FEV_1_. According to a recently published study of 80 women with COPD and 40 controls, not only smoking, but also exposure to biomass combustion, was related to differences in metalloproteinases, including increased MMP-9 and MMP-9/TIMP-1 ratio, among those with COPD [27].

Small observational studies have demonstrated increased MMP-9 in COPD compared to controls, both in analyses of sputum [28], lung parenchyma [8] and serum [10]. To the best of our knowledge, this is the first study, in which serum-MMP-9 has been analysed and also proven to be increased in a population-based COPD-cohort. Furthermore, the association between serum MMP-9 and impaired lung function, assessed as FEV_1_, in COPD show that MMP-9 is related to disease severity which could indicate that MMP-9 is involved in the disease process in COPD. TIMP-1 is suggested to inhibit the proteolytic activity of MMPs [11,12], and the observed association between overweight and TIMP-1 may imply a protecting mechanism by overweight in COPD. We also confirmed, as previously shown [29,30], that serum TIMP-1 is associated with age. The MMP-9/TIMP-1 ratio was analysed in order to evaluate the protease-antiprotease imbalance of possible importance in the pathogenesis of COPD and, besides increasing pack years, decreasing FEV_1_% predicted was close to significantly related to the ratio.

Chronic bronchitis is a well-known predictor of accelerated lung function decline and increased risk for exacerbations in COPD [31,32]. Also less longstanding bronchitis symptoms, such as productive cough, seem to be an important prognostic marker in COPD, accelerating the rate of decline in lung function [33], increasing the risk for exacerbations [34] and mortality [33]. In a cohort of 100 COPD-patients recruited from outpatient care, sputum MMP-9 levels were associated with the proportion of sputum neutrophils, independent of age, prior smoking and presence of airflow obstruction [28]. We found a positive association between productive cough and serum MMP-9 in COPD, indicating increased proteolytic activity in COPD patients with bronchitis, even though this association did not reach statistical significance in the multivariate model. Today, COPD is considered an inflammatory lung disease with systemic effects which, in the western society, is caused primarily by tobacco smoke exposure [28,35] and a population-based study will include subjects with different clinical manifestations or phenotypes of COPD. In the present population-based study, the observed biomarker signals are thus important to note, as they are clearly hypothesis-generating for further studies. The association between increased MMP-9 and productive cough among subjects with COPD shown here contribute, together with previous reports from selected study populations [9,36,37], to the hypothesis that MMP-9 may be involved in the disease process in COPD-subjects with bronchitis symptoms and a history of frequent exacerbations, or the ‘exacerbating-bronchitis’ phenotype [38]. However, in a cross-sectional study such as ours, only associations can be evaluated and longitudinal studies are important for the understanding of MMP-9 and TIMP-1 in relation to measures of disease progression and pathogenesis of different phenotypes of COPD.

Other clinical manifestations of COPD to which MMP-9 may be of importance in the pathogenesis are emphysema and rapid decline in lung function. It has previously been suggested that MMP-9 is related to the development of emphysema [39-41]. In a one-year follow up of 126 patients with the more homogenous phenotype of COPD related to alpha_1_-antitrypsin deficiency, plasma MMP-9 was associated not only with more frequent exacerbations, but also with a decline in transfer factor and a reduction in lung density assessed by CT scan (computer tomography) [35]. In several studies including COPD populations with different clinical phenotypes, MMP-9 has been related to lower lung function [10,27,35,42] and, in our study, we have shown that the relationship between MMP-9 and lower lung function, assessed as FEV_1_% predicted, also applies to COPD in a population-based cohort, dominated by mild to moderate disease; GOLD stages 1–2.

Assessment of COPD disease severity is most often based on the GOLD spirometric classification divided into stages 1–4 [1]. Each stage includes rather wide ranges of FEV_1_% predicted and, thus, the use of FEV_1_% predicted, as a continuous variable, may be more sensitive when it comes to evaluating biomarkers related to disease severity. Our results illustrated this phenomenon fairly well, as decreasing FEV_1_% predicted, but not GOLD stages, was associated with MMP-9 and MMP-9/TIMP-1 ratio among subjects with COPD. In epidemiological studies identifying COPD by spirometry, the GOLD 1 group will include a non-negligible proportion of non-smokers without respiratory symptoms, especially among elderly [43]. Thus the GOLD 1 group may include some “noise” that has to be taken into account when interpreting data.

The strength of this study is the large population-based COPD cohort identified by standardized spirometry, and with a distribution of disease severity conforming well to other population-based studies [9,44]. Thus, both the internal and external validity are considered good, and the study contributes with novel data that imply that MMP-9 is related to disease severity and symptoms in not only selected COPD populations but also population-based cohorts. However, there are also limitations of the study that merit some discussion. First, the ELISA used to measure the levels of MMP-9 and TIMP-1, include both total and pro-enzyme levels. Second, it has been suggested that serum MMP-9 levels do not reflect overall MMP-9 airway activity, as COPD is associated with higher levels of MMP-9 but not with increased MMP-9 activity. Further, the samples were stored several years before the analyses were performed, and it has been shown that the level of these biomarkers decrease by time [45]. However, the geometric decrease in measurable enzyme should be the same for all samples; half-life for degradation is identical for a single enzyme and the possible effects of storage are expected to affect all samples in an equivalent manner. We can exclude another potential source of error, namely the use of different ELISA-kit-batches when performing analyses at different time-points. Thus the measured absolute values may be influenced by storage, but not likely the results, regarding the observed correlations. In the present study, the MMP-9/TIMP-1 ratio was calculated based on the concentrations. This way of addressing the systemic protease-anti-protease balance has been employed in a number of recent studies [43,46,47]. A biologically more relevant way of estimating the proteolytic balance may also include the molar MMP-9/TIMP-1-ratio that has been used in some studies [43]. However, as only the total MMP-9 concentration was determined here, no data on active MMP-9 are available and, hence, a relevant molar ratio cannot be calculated. This fact is a limitation of the study and analyses of both the active and total MMP-9 concentrations and their relation to TIMP-1 are warranted in future studies.

Another factor to be considered is that the fixed ratio of (FEV_1_/FVC <0.70) carries a risk of COPD over-diagnosis in elderly and of under-diagnosis in younger [43]. However, the OLIN COPD study was designed after the shift of the millennium just after the launch of the GOLD guidelines recommending this fixed ratio for the definition of airway obstruction. If a similar epidemiological study were to be designed today, the LLN (lower limit of normal) definition of airway obstruction would be considered.

## Conclusion

This is the first population-based cross-sectional study analysing and demonstrating that serum MMP-9 is higher in COPD compared with in non-COPD. Productive cough and decreasing FEV_1_ were each associated with MMP-9 among subjects with COPD, and decreasing FEV_1_ remained significantly associated with MMP-9 also after adjustment for common confounders in this population-based COPD cohort. The MMP-9/TIMP-1-ratio may suggest an imbalance in the pulmonary protease-antiprotease homeostasis in COPD, with a predominance of proteolytic activity that is related to impaired lung function and, thus, disease severity. Future longitudinal studies are important for the further understanding of MMP-9 and TIMP-1 in relation to disease progress and the pathogenesis of different COPD phenotypes.
